# APPLYING THE DYNAMIC SUSTAINABILITY FRAMEWORK TO AN INTERGENERATIONAL TECHNOLOGY PROGRAM IN HIGHER EDUCATION

**DOI:** 10.1093/geroni/igae098.0703

**Published:** 2024-12-31

**Authors:** Jill Juris, Rachel Scrivano, Skye Leedahl, Meaghan Colvin, Josie Santilli, Shannon Jarrott

**Affiliations:** Appalachian State University, Boone, North Carolina, United States; Boston College, Chestnut Hill, Massachusetts, United States; University of Rhode Island, Kingston, Rhode Island, United States; University of Rhode Island, Kingston, Rhode Island, United States; University of Rhode Island, Kingston, Rhode Island, United States; The Ohio State University, Columbus, Ohio, United States

## Abstract

Cyber-Seniors (CS) is an intergenerational technology program developed to increase older adults’ digital competence and adoption through reverse-mentoring facilitated by trained students. Leveraging higher education as a platform offers mutual benefits, fostering positive attitudes toward aging among students while enhancing technology literacy among older adults. CS often operates within community settings with volunteers and high school students; however, systematic assessment within higher education remains unexplored. This study examined the determinants of sustainability associated with implementing CS in higher education through the Dynamic Sustainability Framework. Through semi structured interviews conducted between April and October 2023, we explored challenges and facilitators employed by ten partnered higher education institutions and two key CS representatives. Data were analyzed by two coders using directed qualitative content analysis in Atlas.ti. Three themes characterized higher education and CS representative experiences implementing CS: adapting the intervention for higher education, navigating the role of resources, and engaging university and community buy-in. The COVID-19 pandemic introduced unique challenges to CS implementation. This study presents the first comprehensive evaluation of CS in higher education, highlighting the necessity of tailored approaches and identifying barriers to sustainability. Results suggest the need for a structured implementation checklist to optimize program delivery within higher education.

